# Pharmacokinetics, Pharmacodynamics and Bioavailability of ACM‐001.1 (*S*‐Pindolol Benzoate) in Healthy Volunteers

**DOI:** 10.1002/jcsm.13651

**Published:** 2024-12-12

**Authors:** Frank Misselwitz, Dennis Henderson, Somasekhara R. Menakuru, Elaine Morten, Chris Roe, Gareth Whitaker, Stefan Wohlfeil, John McDermott

**Affiliations:** ^1^ Actimed Therapeutics London UK; ^2^ Quotient Sciences Nottingham UK

**Keywords:** ACM‐001.1, bioequivalence, cancer cachexia, pharmacodynamics, pharmacokinetics, stereoconversion

## Abstract

**Background:**

*S*‐pindolol has metabolic effects of potential benefit in cancer cachexia: reduced catabolism through nonselective β‐blockade; increased anabolism through partial β2 receptor agonism; and increased appetite and reduced fatigue through central 5‐hydroxytryptamine/serotonin receptor activity. A Phase 2a clinical trial demonstrated that *S*‐pindolol can reverse weight loss and improve fat‐free mass in patients with cancer‐related weight loss. A comparative phase I bioavailability study of *S*‐pindolol and racemic pindolol was performed to support the development of *S*‐pindolol in cancer cachexia.

**Methods:**

This two‐part study assessed the comparative bioavailability and pharmacokinetics of single doses of *S*‐pindolol benzoate (ACM‐001.1) or pindolol (Part 1) and the steady‐state pharmacokinetics and pharmacodynamics of multiple doses of ACM‐001.1 and pindolol (Part 2) in healthy volunteers (NCT06028321). ACM‐001.1 5, 10 and 15 mg and pindolol 15, 20 and 30 mg were tested. In Part 1, subjects were randomised to ACM‐001.1 15 mg followed after a 48‐h washout period by pindolol 30 mg, or the reverse sequence; another group received pindolol 15 mg. Subjects in Part 2 were randomised to pindolol 20 mg twice‐daily or ACM‐001.1 5, 10 or 15 mg twice‐daily for 4 days. Bioavailability, pharmacokinetics, pharmacodynamics, potential for and extent of stereoconversion, and tolerability were assessed.

**Results:**

Parts 1 and 2 included 24 and 27 healthy volunteers, respectively. ACM‐001.1 had predictable pharmacokinetics up to a dose of 15 mg twice daily, with low intersubject variability, after single and multiple doses (*T*_max_ 1 vs. 1.5 h; *C*_max_ 74 vs. 73.6 ng/mL; AUC_(0−*t*)_ 440 vs. 414 ng·h/mL; *t*_1/2_ 4.042 vs. 3.566 h). The bioavailability of *S*‐pindolol after equivalent doses of pindolol (20 mg) and ACM‐001.1 (10 mg) was comparable, and formal bioequivalence margins were met (90% CI for *C*_max_, AUC_(0−*t*)_ and AUC_(0–inf)_ within 80%–125% bioequivalence acceptance criteria). No evidence of stereoconversion of the *S*‐enantiomer into the *R*‐enantiomer, no accumulation, dose linearity and dose proportionality of *S*‐pindolol over a range of doses were demonstrated; we also show indirectly that there was no food effect. ACM‐001.1 was generally well tolerated, with no apparent relationship of side effects to dose, no serious adverse events, severe treatment‐emergent adverse events (TEAEs) or deaths, and similar incidences of TEAEs (fatigue, dizziness, somnolence, nausea and headache) with ACM‐001.1 10 and 15 mg and pindolol 20 mg.

**Conclusions:**

Data from this bridging study of enantiomerically pure ACM‐001.1 and its parent racemic drug, pindolol, support clinical trials of ACM‐001.1 for the treatment of cancer cachexia.

## 1 Introduction

Cachexia is a multifactorial syndrome defined by an ongoing loss of skeletal muscle mass (with or without loss of fat mass) that cannot be fully reversed by conventional nutritional support and leads to progressive functional impairment. Its pathophysiology is characterised by a negative protein and energy balance, driven by a variable combination of reduced food intake and abnormal metabolism [1]. Cancer cachexia shares many of the neurohormonal, biochemical and inflammatory features common to all forms of cachexia, which together result in increased catabolism, decreased anabolism and increased inflammation, the key physiological components of cachexia [2].

Pindolol, a racemic mixture of *R*‐ and *S*‐enantiomers, is a nonselective synthetic β‐adrenergic receptor blocking agent (β‐blocker) with high intrinsic sympathomimetic activity (ISA) [3] that is used to treat hypertension and angina at doses of up to 45–60 mg/day [4]. In addition to its β‐blocking activity, pindolol is a potent antagonist of 5‐hydroxytryptamine/serotonin (5HT1a) receptors and binds to 5HT1a receptors in the brain [5]. Both its β‐blocking and 5HT1a activities reside primarily in the *S*‐enantiomer, whereas ISA is shared equally by the *R*‐ and *S*‐enantiomers [5, 6]. Evidence suggests preferential distribution of the *S*‐enantiomer into the central nervous system, where it has higher affinity for the presynaptic site and may have a partial agonist effect [7]. In animal models, *S*‐pindolol has been shown to augment and decrease the time to onset of selective serotonin reuptake inhibitor therapy via blockade of 5HT1a receptors [7]. In addition, *S*‐pindolol shows greater ventricular β‐adrenoceptor blockade and lower ISA than an equivalent dose of *R*‐pindolol. Furthermore, the *R*‐ and *S*‐enantiomers of pindolol differ in both their pharmacokinetics and pharmacodynamics: the *S*‐enantiomer has a 30% higher rate of tubular secretion than the *R*‐enantiomer [8], and the area under the plasma concentration–time curve (AUC), cumulative urinary excretion (Ae_∞_) and half‐life (*t*_1/2_) of the *S*‐enantiomer have been reported to be greater than those of the *R*‐enantiomer [9].

*S*‐pindolol was the lead candidate from a programme to assess and optimise the potential benefit of racemic β‐blockers for cachexia. It has multifunctional effects relevant for cancer cachexia: reduces catabolism through nonselective β‐blockade [6, 10, 11]; increases anabolism through partial β2 receptor agonism [12]; and increases appetite and reduces fatigue through central 5HT1a activity [11, 13]. *S*‐pindolol has been shown to prevent tissue wasting, improve survival and improve cardiac function in animal models of cancer cachexia [13, 14, 15]. A phase 2a proof of concept clinical study in patients with non–small cell lung cancer or colorectal cancer and weight loss showed that *S*‐pindolol significantly reversed weight loss, improved fat free mass, maintained fat mass and improved handgrip strength [11, 16]. Furthermore, the safety profile of pindolol is well characterised [16, 17], and it is considered that the safety profile, contraindications and precautions for *S*‐pindolol when used in patients with cachexia are likely to be similar to those established for the racemic product. Based on improved stability characteristics, the benzoate salt of *S*‐pindolol (ACM‐001.1) was selected for development for the treatment of cancer cachexia. In vivo, *S*‐pindolol benzoate dissociates fully, resulting in the active pharmacological moiety, *S*‐pindolol.

Bridging from enantiomerically pure drugs to parent racemic drug requires assessing the comparative bioavailability of the single enantiomer and the corresponding enantiomer in the racemic mixture, as well as demonstrating a lack of in vivo stereoconversion [18]. This phase I study assessed the bioavailability, pharmacokinetics, pharmacodynamics, potential for and extent of stereoconversion, and safety and tolerability of single and multiple doses of ACM‐001.1 compared with racemic pindolol in healthy subjects.

## 2 Methods

### 2.1 Study Design

This was a two‐part study to assess the comparative bioavailability and pharmacokinetics of a single dose of ACM‐001.1 and two single doses of pindolol (a racemic mixture of *S*‐ and *R*‐pindolol enantiomers) (Part 1) and to evaluate the steady‐state pharmacokinetics and pharmacodynamics of ACM‐001.1 (Part 2) in healthy volunteers (NCT06028321).

Part 1 was a part‐blinded, part‐randomised, partial cross‐over study. A total of 24 subjects were to be randomised into one of two groups (Group 1, *n* = 16; Group 2, *n* = 8) to ensure evaluable data for at least 14 subjects. Group 1 was double‐blinded and randomised to ACM‐001.1 followed by pindolol or the reverse sequence to evaluate comparative bioavailability in a two‐period cross‐over. Group 2 was open‐label, nonrandomised and assessed the pharmacokinetics of pindolol at a lower dose than used in Group 1 in a single period.

Part 2 was a subject‐blinded, randomised, parallel‐group, dose ranging, multiple‐dose study with a comparator in healthy subjects. It was planned to enrol 32 subjects who were randomised to one of four regimens (*n* = 8 each) to ensure evaluable data in at least six subjects per regimen.

This study was conducted by Quotient Sciences, Nottingham, UK, in accordance with International Council for Harmonisation Good Clinical Practice (GCP) including the integrated Addendum E6, Medicines for Human Use (Clinical Trials) Regulations [19, 20, 21, 22, 23], and Declaration of Helsinki and its amendments [24]. Approvals from the ethics committee and the UK Medicines and Healthcare products Regulatory Agency were obtained. Monitoring and pharmacovigilance were performed by Bionical EMAS (Hitchin, UK).

### 2.2 Subjects

Subjects were healthy male and nonpregnant, nonlactating healthy female volunteers of 20–45 years of age with a body mass index of 18.0–30.0 kg/m^2^ and weight of 50–100 kg at screening who agreed to adhere to contraception requirements (full eligibility requirements are shown in Supporting Information). All subjects provided written informed consent.

### 2.3 Treatment

Doses of ACM‐001.1 of 5, 10 and 15 mg and of pindolol of 15, 20 and 30 mg were selected; these are within the approved dose levels for pindolol [25, 26]. No up‐titration or tapering of single doses of pindolol was proposed, based on a phase I study in healthy subjects of nine consecutive 15 mg doses at 8 hourly intervals (total daily dose 45 mg) [27]. According to the VISKEN (pindolol) Product Monograph [4] and Schwarz [27], pindolol doses up to 20 mg behave in a dose‐linear fashion. Dose linearity, however, is not described and is unknown for higher doses, for example, 30 mg. Therefore, dose linearity for the higher dose of pindolol 30 mg needed to be established in this study to enable a full dose‐range comparison.

ACM‐001.1 was manufactured and provided as 5‐mg tablets by Custom Pharma Services (Hove, UK). Pindolol (VISKEN) was manufactured by Phoenix Labs, Clonee, County Meath, Ireland, and provided as 5‐mg tablets. ACM‐001.1 placebo tablets were provided by Custom Pharma Services.

The treatment regimens used in Parts 1 and 2 are shown in Table 1.

**TABLE 1:** Test regimens.

Regimen	Treatment	Dose	Route of administration
Part 1			
Group 1, regimen A	ACM‐001.1 and placebo	15 mg ACM‐001.1 (as 3 × 5‐mg tablets) plus 3 placebo tablets	Oral, fasted
Group 1, regimen B	Pindolol	30 mg (as 6 × 5‐mg tablets)	Oral, fasted
Group 2	Pindolol	15 mg (as 3 × 5‐mg tablets)	Oral, fasted
Part 2			
Regimen D	Pindolol	20 mg (as 4 × 5‐mg tablets) twice‐daily for 4 days^a^	Oral, fed^b^
Regimen E	ACM‐001.1 and placebo	5‐mg ACM‐001.1 (as 1 × 5‐mg tablet) plus 3 placebo tablets twice‐daily for 4 days^a^	Oral, fed^b^
Regimen F	ACM‐001.1 and placebo	10‐mg ACM‐001.1 (as 2 × 5‐mg tablets) plus 2 placebo tablets twice‐daily for 4 days^a^	Oral, fed^b^
Regimen G	ACM‐001.1 and placebo	15‐mg ACM‐001.1 (as 3 × 5‐mg tablets) plus 1 placebo tablet twice‐daily for 4 days^a^	Oral, fed^b^

^a^ Administered twice daily on Days 1–3 and on the morning of Day 4.

^b^ Subjects were provided with a standardised menu.

### 2.4 Procedures

Subjects were screened for enrolment up to 28 days before dosing and were admitted the evening of the day before dosing (Day −1). In Part 1, subjects in both groups received their initial regimen on the morning of Day 1 following a minimum 10‐h fast and received a standard breakfast 1‐h post‐dose. Subjects in Group 1 were randomised to one of two treatment sequences, ACM‐001.1 15 mg followed by pindolol 30 mg or the reverse sequence (Table 1), and received their initial regimen on the morning of Day 1. After a washout period of at least 48 h, during which they remained at the clinical unit, subjects received the second regimen and were then discharged 24 h post‐dosing. Subjects in Group 2 were treated with pindolol 15 mg and discharged from the clinical unit 24 h post‐dose. A poststudy follow‐up call took place 2–4 days post‐final dose to ensure the ongoing well‐being of the subjects.

Blood and urine samples for pharmacokinetic analysis were taken at the timepoints shown in Table S1. Analysis of *S*‐pindolol and *R*‐pindolol in plasma was performed at Alderley Analytical Limited (Macclesfield, UK). Plasma and urine concentrations of pindolol were calculated by Quotient Sciences (Nottingham, UK) at each pharmacokinetic assessment timepoint as a summation of *R*‐pindolol and *S*‐pindolol concentrations.

Subjects in Part 2 were randomised to one of four groups (Table 1) and were dosed with pindolol or ACM‐001.1 twice daily (approximately 12 h apart) for 4 days, that is, the morning and evening of Days 1–3 and the morning of Day 4. Each morning and evening dose occurred 30 min and 1 h after the start of standard breakfast or standard evening meal, respectively. Subjects were discharged on the morning of Day 7 after a 3‐day observation period following the final dose to monitor safety. A follow‐up call took place 5–7 days post‐final dose (Day 9–11) to ensure the ongoing well‐being of the subjects.

Blood and urine samples for pharmacokinetic analysis in addition to blood samples and heart rate and blood pressure measurements for pharmacodynamic analysis were taken at the timepoints shown in Table S1. Exploratory pharmacodynamic analyses included DHEA/cortisol, myostatin, folistatin, IGF1, PIIINP, MIG/CXCL9 (leptin), ENA78, ghrelin, GHRH and somatostatin. Respiratory function was assessed in Part 2 pre‐dose on Day 1 and 2 h post‐dose on Day 4 using forced expiratory spirometry.

Safety (adverse events [AEs], laboratory evaluations, vital signs, electrocardiogram (ECG) and body weight) was monitored throughout in both parts of the study. AEs were assessed as mild, moderate or severe and serious AEs (SAEs) were defined as described in the Supporting Information.

Data management was performed by Quotient Sciences using a validated eCRF database system (TrialOne v4.3.8) and was subjected to data consistency and validation checks. Clinical laboratory evaluation was performed by The Doctors Laboratory (London, UK).

### 2.5 Objectives and Endpoints

The primary objectives of Part 1 were as follows: assess the comparative bioavailability of *S*‐pindolol following a single dose of ACM‐001.1 or pindolol; assess the comparative pharmacokinetics in plasma of a single dose of ACM‐001.1 or pindolol; and explore the possible stoichiometric dose relationship in plasma between ACM‐001.1 and *S*‐pindolol when administered as a racemic mixture. Secondary objectives were to assess the comparative pharmacokinetics in urine of a single dose of ACM‐001.1 versus pindolol, to investigate the extent of in vivo stereoconversion of ACM‐001.1 in plasma, and to evaluate the safety and tolerability of ACM‐001.1 (also secondary objectives of Part 2).

The primary objectives of Part 2 study were to characterise steady‐state pharmacokinetics in plasma following multiple doses of ACM‐001.1 and pindolol and steady‐state pharmacodynamics of ACM‐001.1 via exploratory measurements of cardiovascular vital parameters and biomarkers of catabolism/anabolism. Characterisation of steady‐state pharmacokinetics in urine following multiple doses of ACM‐001.1 and pindolol was a secondary objective.

### 2.6 Statistical Analysis

Analysis populations are defined in the Supporting Information. Subjects in Part 1 were considered evaluable if pharmacokinetic data to 24 h post‐dose were available; subjects in Part 2 were considered evaluable if they had pharmacokinetic and pharmacodynamic data to 72 and 48 h post‐final dose, respectively.

Pharmacokinetic parameters for *S*‐pindolol, *R*‐pindolol and pindolol in plasma were estimated using noncompartmental analysis methods in Phoenix WinNonlin software (v8.3, Certara USA). Plasma concentration data and pharmacokinetic parameters were summarised in SAS.

Formal statistical analysis of relative bioavailability of ACM‐001.1 versus *S*‐pindolol (Parts 1 and 2) was performed on *C*_max_, AUC_(0−*t*)_ and AUC_(0−inf)_. The pharmacokinetic parameters underwent a natural logarithmic transformation and were analysed using a mixed effect model with fixed terms for regimen, sequence and period and a random subject within sequence term. For the relative bioavailability assessment between the lower pindolol dose and ACM‐001.1, pharmacokinetic parameters underwent a natural logarithmic transformation and were analysed using an analysis of variance (ANOVA) model with a fixed term for regimen.

Formal statistical analysis of dose proportionality between the two pindolol doses (pindolol 30 mg vs. pindolol 15 mg), pindolol and *S*‐pindolol analytes separately (Part 2) between subjects was performed on *C*_max_/D, AUC_(0−*t*)_/D and AUC_(0−inf)_/D. These dose‐adjusted pharmacokinetic parameters underwent a natural logarithmic transformation and were analysed using an ANOVA model with a fixed term for regimen. Adjusted geometric mean ratios (GMRs) and 90% confidence intervals (CIs) for the comparison between pairwise treatment comparisons were calculated.

For supine systolic BP, diastolic BP and heart rate, summary statistics were calculated, including for changes from baseline (Day 1, pre‐dose) at each time point.

## 3 Results

### 3.1 Patient Demographics and Disposition

A total of 24 subjects (14 females, 10 males; 20–43 years; BMI 19.7–29.9 kg/m^2^) were randomised in Part 1 on 26 November 2021, and 27 were randomised in Part 2 (11 females, 16 males; age 23–43 years; BMI 19.8–29.2 kg/m^2^) between 13 January and 2 June 2022 (Table 2). Twenty‐one subjects completed Part 1; one withdrew consent after receiving one planned dose, one withdrew due to an AE of hypotension after one planned dose, and one withdrew after receiving both doses due to a positive COVID‐19 test result 10 h post‐dose. All 24 subjects received at least one dose of study drug and were included in the safety and pharmacokinetics populations. All 27 subjects in Part 2 completed the study and were included in the safety, pharmacokinetic and pharmacodynamic analysis sets.

**TABLE 2:** Subject demographics (safety population).

	Part 1				Part 2				
	ACM‐001.1 followed by pindolol (*n* = 8)	Pindolol followed by ACM‐001.1 (*n* = 8)	Pindolol 15 mg (*n* = 8)	All (*n* = 24)	Pindolol 20 mg (*n* = 7)	ACM‐001.1 5 mg (*n* = 7)	ACM‐001.1 10 mg (*n* = 6)	ACM‐001.1 15 mg (*n* = 7)	All (*n* = 27)
Median age, years (range)	36.5 (20–43)	29.5 (21–38)	36.0 (25–43)	34.5 (20–43)	30.0 (23–35)	32.0 (23–42)	28.5 (24–42)	33.0 (25–43)	30.0 (23–43)
Race, *n* (%)									
Asian	1 (12.5)	1 (12.5)	1 (12.5)	2 (12.5)	0	1 (14.3)	1 (16.7)	1 (14.3)	3 (11.1)
Black/African American	1 (12.5)	0	0	1 (4.2)	0	0	0	1 (14.3)	1 (3.7)
White	6 (75)	7 (87.5)	7 (87.5)	20 (83.3)	7 (100)	6 (85.7)	5 (83.3)	4 (57.1)	22 (81.5)
Chinese	0	0	0	0	0	0	0	1 (14.3)	1 (3.7)
Male/female, *n* (%)	6/2 (75/25)	2/6 (25/75)	2/6 (75/25)	10/14 (41.7/58.3)	2/5 (28.6/71.4)	4/3 (57.1/42.9)	6/0 (100/0)	4/3 (57.1/42.9)	16/11 (59.3/40.7)
Median height, cm (range)	171.0 (154–185)	164.5 (162–182)	166.5 (156–182)	167.5 (154–185)	170.0 (163–176)	170.0 (157–182)	181.0 (167–185)	171.0 (154–182)	172.0 (154–185)
Median weight, kg (range)	71.0 (63.9–99.4)	71.6 (53.2–81.8)	65.7 (56.6–87.0)	70.9 (53.2–99.4)	66.8 (59.4–83.6)	72.7 (53.9–82.1)	76.9 (66.1–100)	66.2 (58.8–91.9)	71.5 (53.9–100)
Median BMI, kg/m^2^ (range)	25.6 (23.9–29.0)	24.4 (20.3–29.9)	24.5 (19.7–28.7)	24.8 (19.7–29.9)	24.2 (22.4–28.3)	27.1 (19.8–29.0)	24.8 (20.9–29.2)	24.2 (21.6–28.4)	24.6 (19.8–29.2)

Abbreviation: BMI, body mass index.

### 3.2 *S*‐Pindolol

**FIGURE 1:**
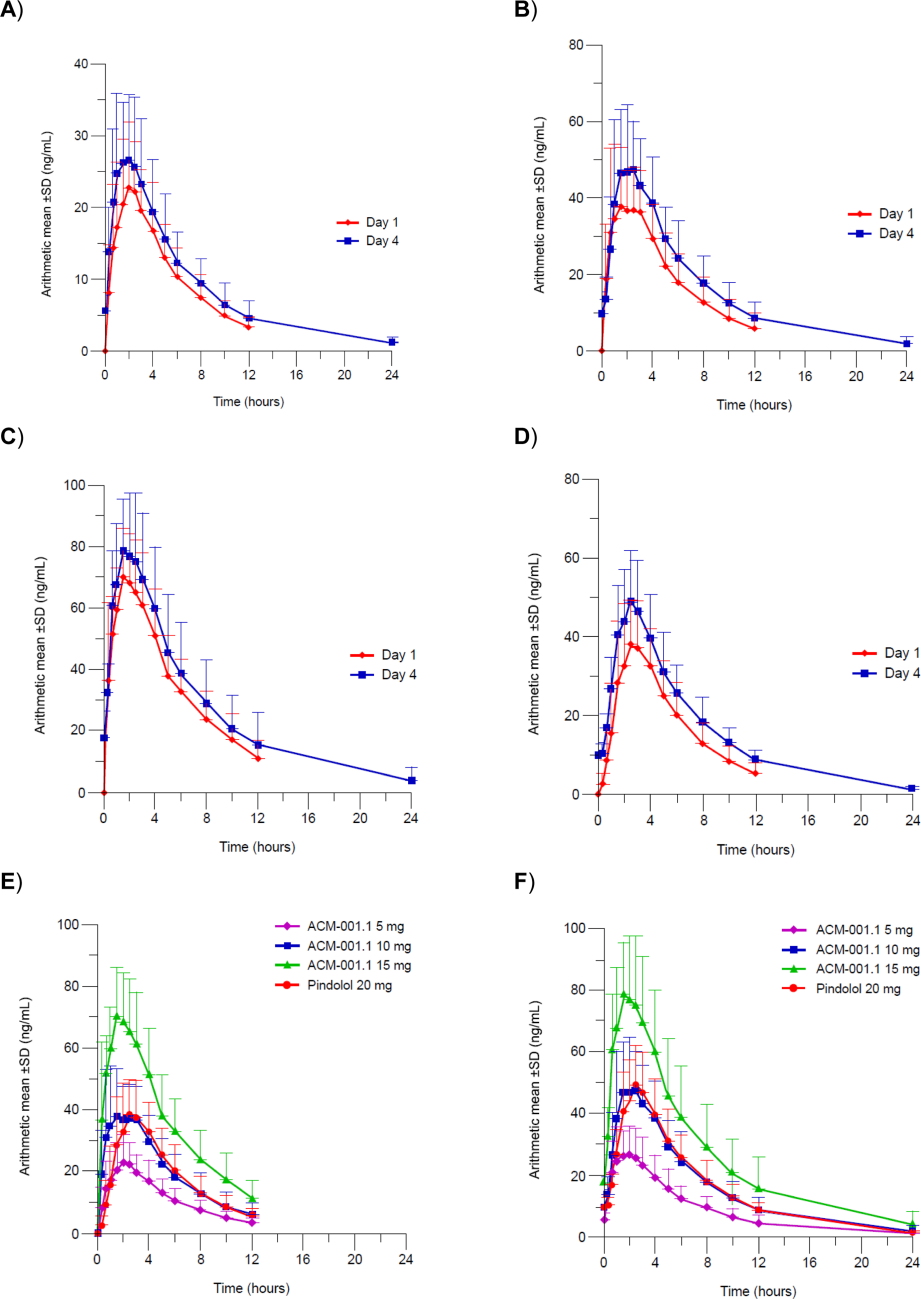
*S*‐pindolol concentrations following single and multiple doses of ACM‐001.1 (A) 5, (B) 10 and (C) 15 mg and of (D) pindolol 20 mg, and relative concentrations of *S*‐pindolol at Days (E) 1 and (F) 4 (Part 2, pharmacokinetic analysis set).

Following single oral doses of ACM‐001.1 5, 10 and 15 mg, and oral doses of racemic pindolol 15, 20 and 30 mg, *S*‐pindolol was rapidly absorbed with median *T*_max_ of 1.00–2.00 h, with no evidence of differences between ACM‐001.1 and pindolol (Table 3). The median *T*_max_ of *S*‐pindolol was similar on Day 4 of twice‐daily dosing of ACM‐001.1 5, 10 and 15 mg and pindolol 20 mg (1.50–2.50 h) (Figure 1; Table 3).

**TABLE 3:** Key plasma pharmacokinetic parameter geometric mean (geometric CV%) values for *S*‐pindolol, *R*‐pindolol and pindolol (A) after a single dose of ACM‐001.1 15 mg or pindolol 15 or 30 mg (Part 1, pharmacokinetic analysis set) and (B) after single and multiple doses of ACM‐001.1 5, 10 or 15 mg or pindolol 20 mg (Part 2, pharmacokinetic analysis set).

(A)							
	*S*‐pindolol			*R*‐pindolol		Pindolol	
	ACM‐001.1 15 mg *n* = 15	Pindolol 15 mg *n* = 8	Pindolol 30 mg *n* = 15	Pindolol 15 mg *n* = 8	Pindolol 30 mg *n* = 15	Pindolol 15 mg *n* = 8	Pindolol 30 mg *n* = 15
*T*_lag_ ^a^ (h)	0.000 (0.00–0.00)	0.000 (0.00–0.32)	0.000 (0.00–0.00)	0.000 (0.00–0.32)	0.000 (0.00–0.00)	0.000 (0.00–0.32)	0.000 (0.00–0.00)
*T*_max_ ^a^ (h)	1.000 (0.33–2.50)	1.250 (0.67–2.10)	2.000 (1.50–5.00)	1.250 (0.67–2.10)	2.000 (1.50–5.00)	1.250 (0.67–2.10)	2.000 (1.50–5.00)
*C*_max_ (ng/mL)	74.0 (25.0%)	39.2 (27.3%)	69.1 (25.2%)	43.9 (29.9%)	75.8 (27.6%)	83.2 (28.6%)	145 (26.1%)
AUC_(0−*t*)_ (ng·h/mL)	440 (35.0%)	225 (37.2%)	414 (33.5%) [*n* = 14]	250 (43.6%)	439 (42.0%) [*n* = 14]	477 (40.0%)	854 (37.6%) [*n* = 14]
AUC_(0−inf)_ (ng·h/mL)	450 (36.8%)	229 (38.3%)	422 (34.6%) [*n* = 14]	256 (43.9%)	448 (43.3%) [*n* = 14]	485 (41.3%)	870 (38.9%) [*n* = 14]
*t*_1/2_ (h)	4.042 (21.7%)	3.917 (19.4%)	3.671 (22.7%) [*n* = 14]	3.858 (22.2%)	3.571 (26.0%) [*n* = 14]	3.829 (24.4%)	3.616 (24.4%) [*n* = 14]
CLr (mL/min)	147 (37.5%)	176 (39.6%)	142 (32.7%) [*n* = 14]	135 (44.7%)	110 (35.0%) [*n* = 14]	155 (42.2%)	123 (33.9%) [*n* = 14]

(B)						
	*S*‐pindolol				*R*‐pindolol	Pindolol
	ACM‐001.1 5 mg *n* = 7	ACM‐001.1 10 mg *n* = 6	ACM‐001.1 15 mg *n* = 7	Pindolol 20 mg *n* = 7	Pindolol 20 mg *n* = 7	Pindolol 20 mg *n* = 7
Day 1						
*T*_max_ ^a^ (h)	2.000 (1.50–2.50)	2.000 (0.67–3.00)	1.500 (0.33–2.50)	2.000 (1.50–5.00)	3.000 (1.50–5.00)	3.000 (1.50–5.00)
*C*_max_ (ng/mL)	23.8 (28.7%)	41.1 (31.8%)	73.6 (19.3%)	40.4 (30.6%)	45.1 (25.7%)	85.5 (27.7%)
AUC_(0−*t*)_ (ng·h/mL)	129 (33.8%)	227 (38.8%)	414 (34.9%)	216 (29.3%)	241 (29.7%)	458 (29.0%)
*t*_1/2_ (h)	3.389 (17.9%)	3.209 (20.6%)	3.566 (23.4%)	2.985 (17.6%)	2.983 (17.6%)	2.994 (21.5)
Day 4						
*T*_max_ ^a^ (h)	1.500 (1.00–2.00)	2.000 (1.00–2.52)	1.500 (0.67–3.00)	2.500 (2.00–3.00)	2.500 (2.00–3.00)	2.500 (2.00–3.00)
*C*_max_ (ng/mL)	27.0 (33.5%)	50.3 (32.9%)	80.5 (27.5%)	48.2 (29.5%)	9.28 (49.4%)	17.6 (40.4%)
C_min_ (ng/mL)	4.27 (75.0%)	7.84 (55.7%)	13.8 (69.5%)	8.26 (31.8%)	54.7 (25.5%)	103 (27.1%)
AUC_(0−*t*)_ (ng·h/mL)	183 (42.7%)	346 (40.5%)	582 (48.4%)	338 (25.2%)	393 (32.0%)	736 (28.4%)
AUC_(0−inf)_ (ng·h/mL)	190 (44.2%)	355 (40.7%)	592 (47.9%)	347 (25.2%)	400 (31.5%)	748 (27.7%)
*t*_1/2_ (h)	4.556 (34.6%)	4.808 (25.3%)	5.426 (26.1%)	4.218 (16.1%)	4.679 (29.5%)	4.490 (23.2%)

Abbreviations: AUC_(0−inf)_, area under the plasma metabolite concentration–time curve from zero to infinity; AUC_(0−*t*)_, area under the plasma concentration–time curve from zero to the time of the last quantifiable concentration; CLr, total clearance; *C*_max_, maximum plasma concentration; *t*_1/2_, terminal half‐life; *T*_lag_, time to first nonzero concentration; *T*_max_, time to maximum concentration.

^a^ Median (range).

The mean peak *S*‐pindolol exposure (*C*_max_) following a single dose of ACM‐001.1 15 mg (74 ng/mL) was slightly higher than that observed after pindolol 30 mg (69.1 ng/mL), with similar overall exposures (AUC_0−inf_) of 450 ng·h/mL and 422 ng·h/mL (Table 3). Similarly, the *C*_max_ of *S*‐pindolol after ACM‐001.1 10 mg and pindolol 20 mg was similar both on Day 1 and Day 4 of twice‐daily dosing; steady state was reached after administration of 5 doses of ACM‐001.1. *C*_max_ values for ACM‐001.1 5 and 10 mg were in proportion to the values observed with ACM‐001.1 15 mg (Figure 1; Table 3). Small CV% values demonstrate that intersubject variability was low throughout the study.

The plasma half‐life of *S*‐pindolol on Day 1 was similar with all doses of ACM‐001.1 tested and with pindolol 15, 20 and 30 mg (Table 3). On Day 4 of twice‐daily dosing, the plasma half‐life of *S*‐pindolol was numerically but not statistically significantly higher than that at Day 1 with ACM‐001.1 5, 10 and 15 mg and pindolol 20 mg (Table 3).

Peak and overall exposures of *S*‐pindolol following a single dose of ACM‐001.1 15 mg were approximately double (89%–96% higher) those with pindolol 15 mg; the lower limit of the 90% CIs of the geometric mean ratios excluded 100% in each case (Table 4). Following a single dose of pindolol 30 mg, generally proportional higher peak and overall exposures were noted for both *S*‐pindolol and pindolol compared with those following pindolol 15 mg (Table 3). Peak and overall *S*‐pindolol exposure measured by *C*_max_, AUC_(0−*t*)_ and AUC_(0−inf)_ for ACM‐001.1 15 mg were statistically comparable to those for 30 mg pindolol (Table 4). Although not a hypothesis, this study was designed to evaluate, *S*‐pindolol exposure following dosing with ACM‐001.1 15 mg can be considered bioequivalent to that of pindolol 30 mg, with the 90% CI for each comparison being wholly contained within the 80%–125% bioequivalence acceptance criteria [28]. Comparing the bioavailability of *S*‐pindolol after multiple doses of ACM‐001.1 10 mg with that after multiple doses of pindolol 20 mg demonstrated that relative bioavailability was maintained over time (Table S2).

**TABLE 4:** Relative bioavailability of *S*‐pindolol following single and multiple doses of ACM‐001.1 and pindolol (Part 1, pharmacokinetic analysis set).

		ACM‐001.1		Pindolol			
Comparison	Pharmacokinetic parameter	*n*	Adjusted geometric mean	*n*	Adjusted geometric mean	Ratio (%)	90% CI
ACM‐001.1 15 mg vs. pindolol 15 mg	*C*_max_ (ng/mL)	15	74.0	8	39.2	188.66	155.85–228.39
	AUC_(0−*t*)_ (ng·h/mL)	15	440	8	225	195.39	150.48–253.70
	AUC_(0−inf)_ (ng·h/mL)	15	450	8	229	196.45	149.68–257.84
ACM‐001.1 15 mg vs. pindolol 30 mg	*C*_max_ (ng/mL)	14	74.5	14	67.7	110.05	103.20–117.36
	AUC_(0−*t*)_ (ng·h/mL)	13	421	13	407	103.57	97.41–110.13
	AUC_(0−inf)_ (ng·h/mL)	13	429	13	415	103.48	97.46–109.88

Abbreviations: AUC_(0−inf)_, area under the plasma metabolite concentration–time curve from zero to infinity; AUC_(0−*t*)_, area under the plasma concentration–time curve from zero to the time of the last quantifiable concentration; *C*_max_, maximum plasma concentration.

Data for the *T*_max_ (1 vs. 1.5 h), *C*_max_ (74 vs. 73.6 ng/mL), AUC_(0−*t*)_ (440 vs. 414 ng·h/mL) and *t*_1/2_ (4.042 vs. 3.566 h) of *S*‐pindolol on Day 1 of the single and multiple dose studies were similar. This suggests that there was no food effect because dosing in the single‐dose study was under fasting conditions and that in the multiple‐dose study was under fed conditions.

*C*_max_/D, AUC_(0−*t*)_/D and AUC_(0−inf)_/D for a dose‐adjusted dose proportionality comparison of *S*‐pindolol following single doses of pindolol 30 mg and 15 mg are presented in Table S3. Dose‐adjusted peak and overall exposure to pindolol and *S*‐pindolol were similar per milligram of drug for pindolol 30 and 15 mg. In each instance, the 90% CI of the geometric mean ratios contained the value 100%, and therefore, the increase in exposure can be considered to be generally proportional with dose.

The renal clearance of *S*‐pindolol appeared higher than that of *R*‐pindolol based on recovery in urine samples. No *R*‐pindolol was recovered in urine after single and multiple doses of ACM‐001.1 in Part 2 of the study (Tables S4 and S5).

### 3.3 *R*‐Pindolol

**FIGURE 2:**
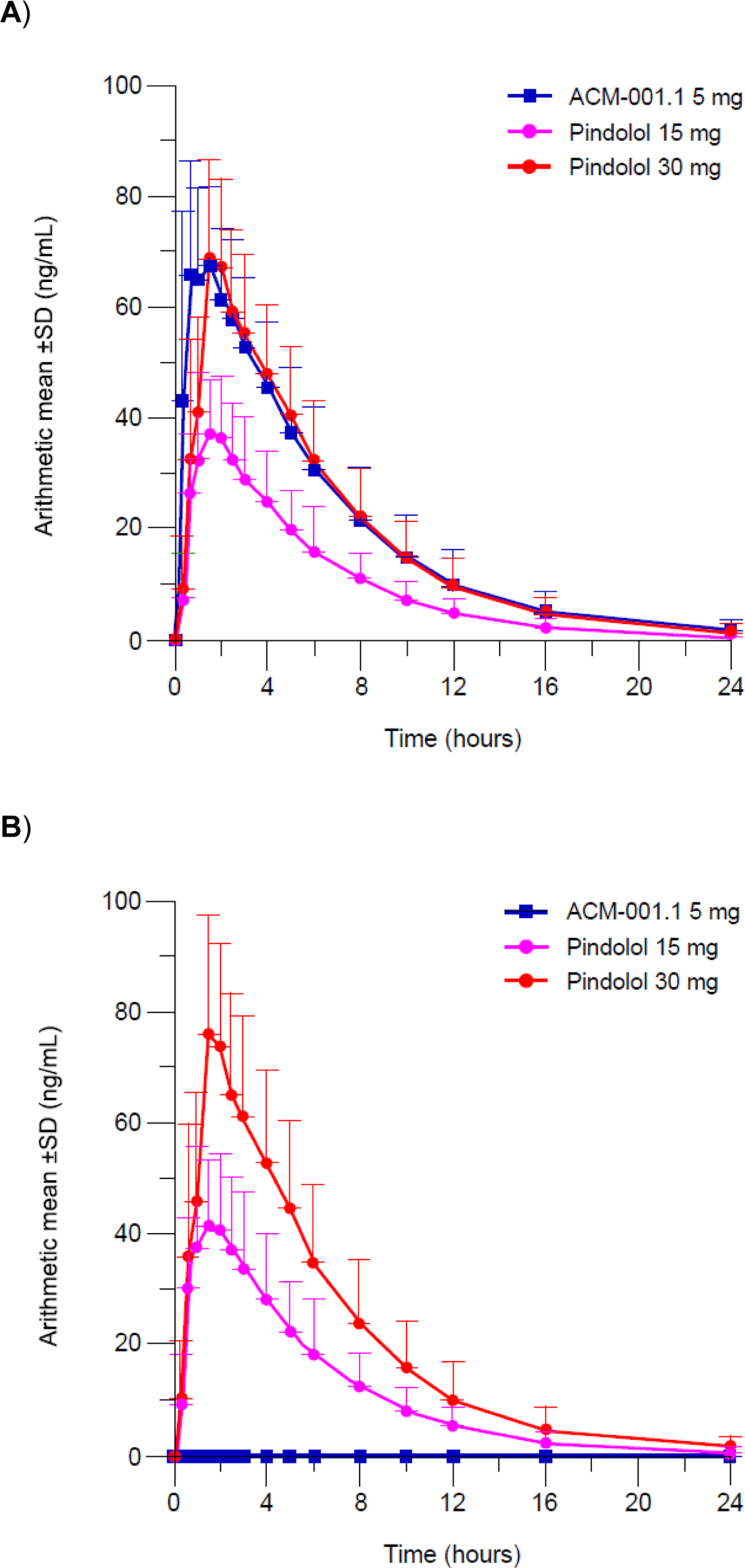
(A) Arithmetic mean *S*‐pindolol and (B) *R*‐pindolol plasma concentrations following a single dose of ACM‐001.1 15 mg, pindolol 15 mg or pindolol 30 mg (Part 1, pharmacokinetic analysis set).

**FIGURE 3:**
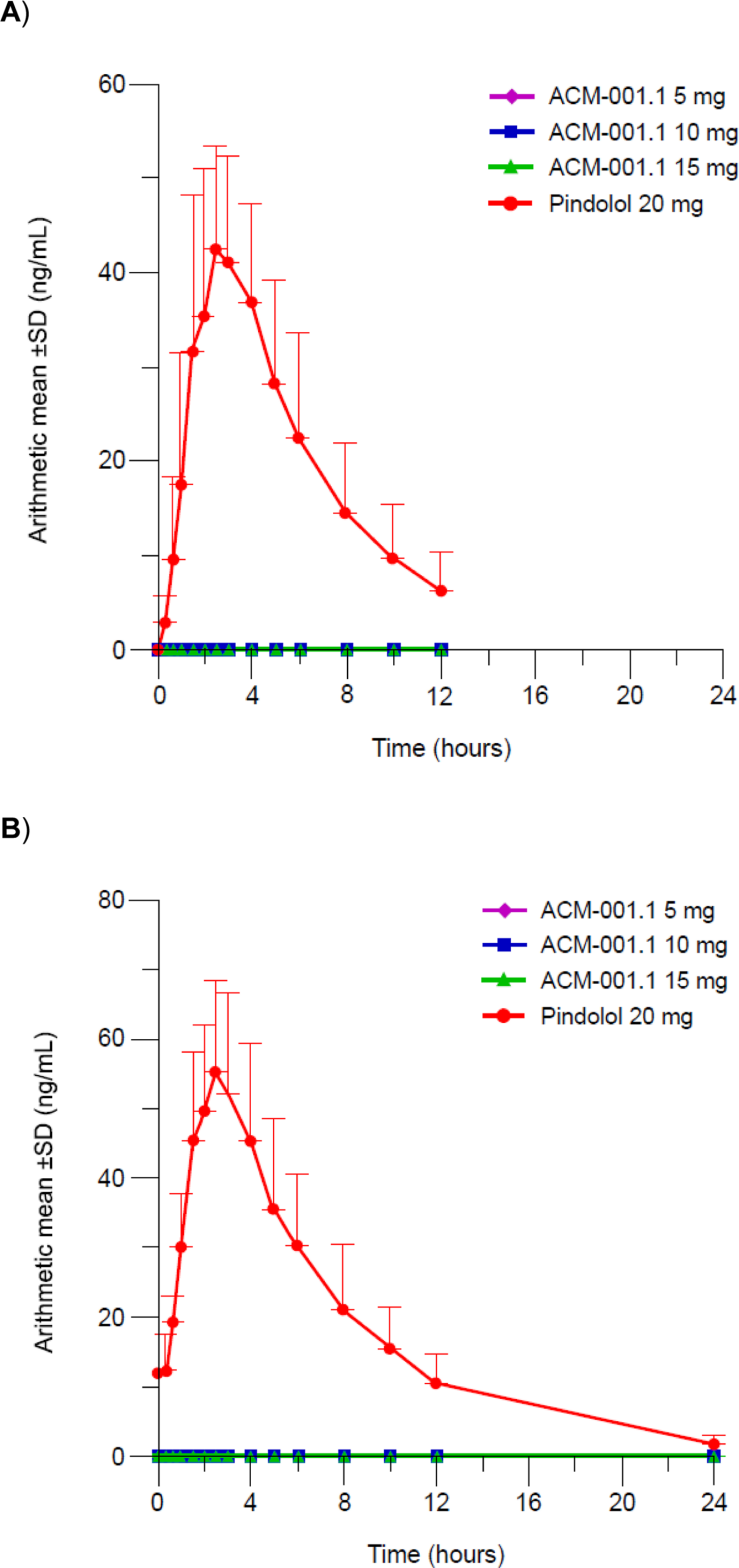
*R*‐pindolol concentrations following (A) single and (B) multiple doses of ACM‐001.1 5, 10 and 15 mg and pindolol 20 mg (Part 2, pharmacokinetic analysis set).

Following administration of a single dose of ACM‐001.1 15 mg, concentrations of *R*‐pindolol in the plasma were not measurable, demonstrating that there had been no conversion of *S*‐pindolol to *R*‐pindolol (Figure 2B). Similarly, no quantifiable concentrations of *R*‐pindolol were observed in the plasma or urine following oral administration of either single or multiple doses of ACM‐001.1 5, 10 or 15 mg (Figure 3). These findings are supported by the results of the analysis of renal clearance.

### 3.4 Safety

Single and multiple doses of ACM‐001.1 5, 10 and 15 mg and of pindolol 15 and 30 mg were found to be generally well tolerated under the conditions of the study. No serious adverse events (SAEs), severe treatment‐emergent adverse events (TEAEs) or deaths were reported.

In total, 49 TEAEs in 19 subjects were reported in Part 1; of these, 37 in 14 subjects were considered possibly related or related to study drug (Table 5). No relationship to dose was apparent. With multiple doses of study drugs in Part 2, the incidences of TEAEs and treatment‐related TEAEs were similar with ACM‐001.1 10 and 15 mg and pindolol 20 mg, but lower with ACM‐001.1 5 mg (Table 5).

**TABLE 5:** Treatment‐emergent adverse events (safety analysis set).

	Part 1				Part 2				
Patients, *n* (%); events, *n*	ACM‐001.1 15 mg (*n* = 15)	Pindolol 30 mg (*n* = 15)	Pindolol 15 mg (*n* = 8)	All (*n* = 24)	ACM‐001.1 5 mg (*n* = 7)	ACM‐001.1 10 mg (*n* = 7)	ACM‐001.1 15 mg (*n* = 6)	Pindolol 20 mg (*n* = 7)	All (*n* = 27)
TEAE	10 (66.7); 19	11 (73.3); 20	5 (62.5); 10	19 (79.2); 49	4 (57.1); 6	6 (100); 20	7 (100); 25	6 (85.7); 25	23 (85.2); 76
Severe TEAE	0	0	0	0	0	0	0	0	0
Related TEAE	6 (40.0); 13	9 (60.0); 15	4 (50.0); 9	14 (58.3); 37	2 (28.6); 2	5 (83.3); 12	7 (100); 21	5 (71.4); 15	19 (70.4); 50
Serious TEAE	0	0	0	0	0	0	0	0	0
TEAE leading to withdrawal	0	1 (6.7);1	0	1 (4.2);1	0	0	0	0	0
TEAE leading to death	0	0	0	0	0	0	0	0	0
Related TEAEs									
Fatigue	3 (20.0); 3	4 (26.7); 4	3 (37.5); 3		0	0	1 (14.3); 1	0	
Dizziness	3 (20.0); 3	3 (20.0); 3	2 (25.0); 2		0	3 (50.0); 5	4 (57.1); 4	2 (28.6); 2	
Somnolence	3 (20.0); 3	2 (13.3); 2	0		0	0	0	0	
Tremor	0	1 (6.7); 1	0		0	0	0	0	
BP decreased	2 (13.3); 2	3 (20.0); 3	1 (12.5); 1		1 (14.3); 1	1 (16.7); 1	2 (28.6); 3	3 (42.9); 6	
Hot flush	0	0	1 (12.5); 1		0	0	0	0	
Nausea	2 (13.3); 2	1 (6.7); 1	2 (25.0); 2		0	0	2 (28.6); 2	1 (14.3); 1	
Vomiting	0	1 (6.7); 1	0		0	0	0	0	
Headache	0	0	0		1 (14.3); 1	1 (16.7); 1	3 (42.9); 4	3 (42.9); 5	
Taste disorder	0	0	0		0	1 (16.7); 1	1 (14.3); 1	0	
ALT increased	0	0	0		0	0	1 (14.3); 1	0	
Palpitations	0	0	0		0	1 (16.7); 1	1 (14.3); 1	0	
Asthenia	0	0	0		0	0	0	1 (14.3); 1	
Muscle spasms	0	0	0		0	1 (16.7); 1	1 (14.3); 1	0	
Rhabdomyolysis	0	0	0		0	1 (16.7); 1	0	0	
Agitation	0	0	0		0	0	1 (14.3); 1	0	
Insomnia	0	0	0		0	0	1 (14.3); 1	0	
Eye pain	0	0	0		0	0	1 (14.3); 1	0	

Abbreviations: BP, blood pressure; TEAE, treatment‐emergent adverse event.

The majority of treatment‐related TEAEs were mild, with the most common being fatigue, dizziness, somnolence, nausea and headache. All of these events resolved by 24 h post‐dose (Table 5). A number of subjects experienced TEAEs of decreases in blood pressure with all three doses of ACM‐001.1 and with pindolol approximately 2 h after dosing, which was approximately concurrent with *T*_max_ (Table 5 and Supporting Information). Of these decreases in blood pressure, only one, with pindolol 30 mg, was assessed as a TEAE of moderate, possibly related hypotension; this led to subject withdrawal. One further subject was withdrawn from the study early after a TEAE of COVID‐19, judged as unrelated to treatment.

After a single dose of ACM‐001.1 or pindolol in Part 1, no clinically important changes in mean haematology or clinical chemistry values from baseline for any dose group were observed, and no individual urinalysis results were considered clinically relevant or reported as TEAEs (Supporting Information). The majority of mean vital sign values were within reference ranges. No clinically relevant differences between ACM‐001.1 15 mg, pindolol 30 mg and pindolol 15 mg for any electrocardiogram parameter and no clinically relevant mean changes from baseline were observed (Supporting Information).

In Part 2, no clinically important changes in the majority of mean haematology or clinical chemistry values from baseline (Day 1, pre‐dose) to any post‐dose time point for any regimen, and no notable differences in mean values between dose levels of ACM‐001.1 or between ACM‐001.1 and pindolol were observed (Supporting Information). However, one subject receiving ACM‐001.1 10 mg had elevated myoglobin (261 IU/L) associated with mild exacerbated foot cramps and elevated creatine kinase (916 IU/L) on Day 4 pre‐dose. A diagnosis of mild rhabdomyolysis possibly related to study drug was made. The TEAE was considered to have resolved at follow‐up. In addition, a subject receiving ACM‐001.1 15 mg experienced mild increased alanine aminotransferase (ALT; 129 IU/L) possibly related to study drug 24 h post‐final dose. Although ALT remained elevated at discharge (127 IU/L), the TEAE was considered to have resolved at follow‐up. The majority of mean vital sign values were within reference ranges. No clinically relevant differences between ACM‐001.1 and pindolol for any electrocardiogram parameter and no clinically relevant mean changes from baseline were observed (Supporting Information).

### 3.5 Pharmacodynamics

Exploratory pharmacodynamic biomarker data are shown in Table S6. No dose‐related trends in changes in pharmacodynamic biomarkers and no differences between ACM‐001.1 and pindolol were noted.

#### 3.5.1 Respiratory Function

In Part 2, there were no notable differences in mean FEV_1_, FVC or FEV_1_/FVC values between regimens, and no notable differences in mean and percentage changes from baseline (Day 1, pre‐dose) for any regimen (Table S7).

#### 3.5.2 Heart Rate

Based on the known effects of pindolol on heart rate [4], we performed a post‐hoc analysis of subject heart rate in Part 2 of the study. This analysis showed that ACM‐001.1 at all doses tested was heart rate neutral, whereas racemic pindolol increased heart rate both on Day 1 and Day 4 (Figure S2).

## 4 Discussion

This study has demonstrated that after single and multiple doses of ACM‐001.1, the benzoate salt of enantiomerically pure *S*‐pindolol, the bioavailability of *S*‐pindolol is similar to that of *S*‐pindolol following administration of an equivalent dose of racemic pindolol. Furthermore, no stereoconversion of *S*‐pindolol to *R*‐pindolol was observed. We have also demonstrated that *S*‐pindolol was rapidly absorbed after single and multiple oral doses of ACM‐001.1 5, 10 and 15 mg and racemic pindolol 15, 20 and 30 mg, with similar *T*_max_. Mean peak concentration (*C*_max_) of *S*‐pindolol was slightly higher after ACM‐001.1 15 mg than pindolol 30 mg, which contain equivalent amounts of *S*‐pindolol, but overall exposure (AUC) was similar, with low intersubject variability, and plasma *t*_1/2_ was unchanged. Based on these observations, *S*‐pindolol exposure following ACM‐001.1 15 mg can be considered to be bioequivalent to that following pindolol 30 mg, with the 90% CI for each comparison being wholly contained within 80%–125% bioequivalence acceptance criteria [28]. In addition, we have demonstrated dose proportionality in terms of peak and overall exposures for ACM‐001.1 at doses up to 15 mg and pindolol at doses up to 30 mg. Taken together, the dose proportionality and bioequivalence of ACM‐001.1 and racemic pindolol and lack of stereoconversion from *S*‐pindolol to *R*‐pindolol support bridging from data for pindolol to ACM‐001.1 [18].

The pharmacokinetic data from the single‐dose study (fasted) and the multiple‐dose study (fed) were similar. This indirectly demonstrates a lack of food effect with ACM‐001.1. This is an important consideration for its use in people with cancer, who have a high incidence of appetite loss [29, 30].

Doses of ACM‐001.1 15 mg and pindolol 15 and 30 mg were selected for use in Part 1 of this study, whereas a pindolol dose of 20 mg twice‐daily was selected for Part 2 of the study; pindolol doses up to 20 mg are known to behave in a dose‐linear fashion [27]. We established dose linearity at a dose of pindolol 30 mg, equivalent to ACM‐001.1 15 mg, in Part 1 of this study. The pindolol doses used were within the recommended dose levels for pindolol, that is, up to a maximum of 45 mg/day given in divided doses twice or three times daily [26].

The safety profile, contraindications and precautions for ACM‐001.1 were expected to be similar to those already established for pindolol, which has been in clinical use for many years and has a well‐established safety profile [26]. Data for TEAEs showed that the tolerability of ACM‐001.1 was generally similar or better than that of an equivalent dose of racemic pindolol. Overall, subjects treated with a single dose of pindolol 30 mg were 50% more likely to experience treatment‐related TEAEs than those treated with a single dose of ACM‐001.1 15 mg (9/15 [60%] and 6/15 [40%] of subjects had events [Table 5]); after multiple doses of ACM‐001.1 10 mg and pindolol 20 mg, the number of treatment‐related TEAEs in the seven subjects in each group was 12 and 15. Specific treatment‐related TEAEs such as fatigue, dizziness, somnolence and nausea tended to occur at similar frequencies with single doses of ACM‐001.1 15 mg and pindolol 30 mg and multiple doses of ACM‐001.1 10 mg and pindolol 20 mg. A number of subjects experienced decreases in blood pressure, a known pharmacological effect of pindolol; however, all treatment‐related events were mild in severity and resolved prior to the end of the study. No significant dose‐related trend in the incidence or type of TEAEs was observed. Furthermore, no negative effects on lung function were observed with either ACM‐001.1 or pindolol. Thus, single and multiple doses of ACM‐001.1 and pindolol were generally well tolerated in this study in healthy volunteers. Similar results were reported in an earlier study in healthy volunteers [31]. In the only reported study to date of the use of *S*‐pindolol (doses of 2.5 mg and 10 mg twice‐daily) to treat and prevent cancer cachexia, treatment was associated with higher rates of anaemia, cough and dyspnoea than placebo [16], with dyspnoea observed predominantly in patients with non–small cell lung cancer, possibly due to the increased likelihood of chronic lung disease in this population [16].

Our data show differences in the effects on heart rate of ACM‐001.1 at doses of 5–15 mg and racemic pindolol 20 mg, with ACM‐001.1 being heart rate neutral whereas racemic pindolol increased heart rate. This is consistent with previous observations that increases in heart rate are observed with both racemic pindolol and *R*‐pindolol, but not *S*‐pindolol [32]. Furthermore, in a study in healthy volunteers, pindolol 0.05 mg/kg increased resting heart rate by 7 bpm [33]. Clark et al. showed that while the ISA is shared by both stereoisomers, only in *S*‐pindolol is its effect countered by beta blockade, which is 100–200‐fold more potent for *S*‐pindolol than *R*‐pindolol [6]. The result is that in *R*‐pindolol, the ISA is not counteracted, leading to heart rate increases. Effects on heart rate are important in patients with cancer cachexia. It has been shown that resting heart rate is significantly higher in patients with colorectal, pancreatic and non–small cell lung cancer than in healthy controls (mean 79 vs. 70 bpm) and that patients with cancer are significantly more likely to have resting heart rate ≥75 and ≥90 bpm [34]. Furthermore, heart rate >75 bpm was found to be a significant independent predictor of mortality [34]. Therefore, the observation that ACM‐001.1 is heart rate neutral suggests that its use to treat cancer cachexia is potentially safer and may be less likely to affect mortality than racemic pindolol.

In conclusion, this study in healthy volunteers has demonstrated that ACM‐001.1 has predictable pharmacokinetics up to a dose of 15 mg twice daily, with low inter‐subject variability after single and multiple doses. The bioavailability of *S*‐pindolol after equivalent doses of pindolol and ACM‐001.1 is comparable and formal bioequivalence margins were met. Furthermore, our data show no evidence of in vivo stereoconversion of the *S*‐enantiomer into the *R*‐enantiomer, no accumulation, dose linearity and dose proportionality of *S*‐pindolol over a wide range of doses, and no food effect. Finally, ACM‐001.1 was generally well tolerated, with TEAEs in line with the expected profile. Together, these data indicate that bridging from enantiomerically pure ACM‐001.1 (*S*‐pindolol benzoate) to the parent racemic drug, pindolol, is appropriate and support further clinical trials of ACM‐001.1 for the treatment of cancer cachexia. Clinical studies of ACM‐001.1 to treat cachexia in patients with colorectal cancer and non–small cell lung cancer have received FDA approval and are being planned [35].

## Ethics Statement

The corresponding author, on behalf of all co‐authors, certifies adherence to the following principles:

- All authors listed on the manuscript have approved its submission and (if accepted) approve publication in the journal
- Each named author has made a material and independent contribution to the work submitted for publication
- No person who has a right to be recognised as an author has been omitted from the list of authors on the manuscript
- The work is original and is neither under consideration elsewhere nor that it has been published previously in whole or in part other than in abstract form
- All authors certify that the work is original and does not contain excessive overlap with prior or contemporaneous publication elsewhere
- All original research work has been approved by the relevant bodies
- All relevant conflicts of interest, financial or otherwise, that may have affected the authors' ability to present data objectively, and relevant sources of funding of the research in question have been declared in the manuscript
- All authors certify that they will submit the original source data to the editorial office upon request
- The manuscript in its published form will be maintained on the servers of the journal as a valid publication only as long as all statements in these guidelines remain true; If any of the aforementioned statements ceases to be true, the authors will notify as soon as possible the Editor‐in‐Chief of the journal, so that the available information regarding the published article can be updated and/or the manuscript can be withdrawn.

## Conflicts of Interest

Frank Misselwitz, Elaine Morten and Stefan Wohlfeil are employees of and hold shares and share options in Actimed Therapeutics, Ascot, UK. John McDermott, Chris Roe, Gareth Whitaker, Dennis Henderson and Somasekhara R Menakuru are employees of Quotient Sciences Limited, Nottingham, UK.

## Supporting information


**Data S1.** Supporting Information

**Figure S1.** Heart rate on Day 4 with racemic pindolol 20 mg and ACM‐001‐1 5, 10 and 15 mg at steady state (Part 2, pharmacodynamic analysis set)
