# Supplementary material for: Pharmacokinetics, Pharmacodynamics and Bioavailability of ACM‐001.1 (S‐Pindolol Benzoate) in Healthy Volunteers
Source: J Cachexia Sarcopenia Muscle. 2024 Dec 12;16(1):e13651. doi: 10.1002/jcsm.13651 (PMC11670174; doi:10.1002/jcsm.13651)

Pharmacokinetics, pharmacodynamics and bioavailability of ACM-001.1 (S-pindolol benzoate) in healthy volunteers

Supplementary materials – methods

Eligibility Criteria

Inclusion Criteria

- Healthy males or non-pregnant, non-lactating healthy females
- Aged 20 to 45 years inclusive at the time of signing informed consent
- Body mass index (BMI) of 18.0 to 30.0 kg/m2 as measured at screening
- Weight of 50 to 100 kg at screening
- Must be willing and able to communicate and participate in the whole study
- Must provide written informed consent
- Must agree to adhere to the contraception requirements defined in the study protocol

Exclusion Criteria

- Subjects who have received any IMP in a clinical research study within the 90 days prior to, or less than 5 elimination half-lives prior to first dosing whichever is longer
- Subjects who are, or are immediate family members of, a study site or sponsor employee
- Subjects for whom pindolol is contradicted: hypersensitivity to the active substance or to any of its listed excipients, untreated cardiac failure, atrioventricular block, cardiogenic shock, pronounced bradycardia, second and third degree heart block, obstructive pulmonary disease, bronchial asthma and history of bronchospasm, history of cor pulmonale, metabolic acidosis, prolonged fasting, severe renal failure, sick sinus syndrome, Prinzmetal angina, untreated phaeochromocytoma, peripheral circulatory disturbances
- Subjects who have previously been administered IMP in this study.
- Evidence of current SARS-CoV-2 infection.
- History of any drug or alcohol abuse in the past 2 years

- Regular alcohol consumption in males >21 units per week and females >14 units per week (1 unit = ½ pint beer, or a 25 mL shot of 40% spirit, 1.5 to 2 units = 125 mL glass of wine, depending on type)
- A confirmed positive alcohol breath test at screening or admission
- Current smokers and those who have smoked within the last 12 months.
- Current users of e-cigarettes and nicotine replacement products and those who have used these products within the last 12 months
- Females of childbearing potential who are pregnant or lactating. Subjects with a positive highly sensitive urine test [at admission] or serum pregnancy test [at screening])
- Male subjects with pregnant or lactating partners
- Subjects who do not have suitable veins for multiple venepunctures/cannulation as assessed by the investigator or delegate at screening
- Clinically significant abnormal clinical chemistry, haematology or urinalysis as judged by the investigator. Subjects with Gilbert's Syndrome are allowed
- Confirmed positive drugs of abuse test result
- Positive serological reaction of syphilis, hepatitis B surface antigen (HBsAg), hepatitis C virus antibody (HCV Ab) or HIV antibody and antigen results
- History of clinically significant cardiovascular (overt cardiac failure, cardiogenic shock, angina or coronary heart disease, peripheral vascular disease, history of blackouts), history of Raynaud's disease or phenomenon, renal, hepatic, dermatological (e.g. psoriasis), chronic respiratory or gastrointestinal disease, neurological or psychiatric disorder (clinically significant history of depression/mood disorder/suicidal ideation or behaviour), as judged by the investigator
- Subjects with clinically significant abnormalities on ECG at screening or first pre-dose including but not confined to heart rate <50 bpm, PR interval >220 msec, QRS duration >120 msec, non-sinus rhythm, left bundle branch block, 2nd or 3rd degree heart block, QTcF >450 msec. ECG can be repeated once if parameters are out of range
- Subjects who are found to have mean heart rate less than 50 bpm at rest or mean systolic blood pressure less than 100 mmHg or mean diastolic blood pressure less than 50 mmHg when taken as the mean of three measurements at least 1 min after the previous one at screening or first pre-dose. One repeat of the triplicate measurements is allowed to confirm if out of range

- Subjects with orthostatic hypotension at screening or first pre-dose as defined by a drop in systolic blood pressure >20 mmHg or diastolic blood pressure of 10 mmHg after standing upright for at least 3 min after being supine for 5 min
- Presence or history of clinically significant allergy requiring treatment, as judged by the investigator. Hay fever is allowed unless it is active
- Donation of blood or plasma within the previous 3 months or loss of greater than 400 mL of blood
- Subjects who are taking, or have taken, any prescribed or over-the-counter drug or herbal remedies (other than up to 4 g of paracetamol per day, HRT/hormonal contraception) in the 14 days before IMP administration (see Section 11.4). Pindolol should not be taken in conjunction with agents which inhibit calcium transport e.g. verapamil. COVID-19 vaccines are accepted concomitant medications. Exceptions may apply on a case-by-case basis, for medication with a short half-life if the washout is such that no pharmacodynamic activity is expected by the time of dosing with IMP; the use of medication does not jeopardize the safety of the trial subject; and if considered not to interfere with the objectives of the study, as determined by the investigator
- Subjects who are labile diabetics with risk of acute hypoglycaemia
- History of adult bronchial asthma
- Subjects with active or dormant psoriasis
- Subjects scheduled for elective surgery or other medical procedures
- Failure to satisfy the investigator of fitness to participate for any other reason

Contraception and restrictions

Male subjects with partners of childbearing potential

Male subjects who are sexually active with a partner of child-bearing potential must use, with their partner, a condom plus an approved method of effective contraception from the time of informed consent until 91 days after last IMP administration. This has been calculated based on 90 days (one cycle of spermatogenesis) plus 5 half-lives of the study drug. Five half-lives has been calculated as 1 day.

The following methods are acceptable:

- Partner's use of combined (oestrogen and progestogen-containing) hormonal contraception associated with inhibition of ovulation:
    - oral
    - intravaginal
    - transdermal
- Partner's use of progestogen-only hormonal contraception:
    - oral
    - injectable/implantable
    - intrauterine hormone-releasing system
- Partner's use of intrauterine device
- Vasectomised
- Partner's bilateral tubal occlusion
- Partner's use of female cap or diaphragm or sponge with spermicide (double barrier)

These contraception requirements are considered to be more conservative than the guidance issued by the Heads of Medicines Agency: Clinical Trials Facilitation Group [1].

Males with partners of non-childbearing potential

There is a significant risk of drug exposure through the ejaculate (which also applies to vasectomised males) that might be harmful to sexual partners. Therefore, even if a male is sexually active with a partner of non-childbearing potential, they will be required to use a condom from first administration of study drug until the follow-up phone call.

Female subjects of childbearing potential

Female subjects who are sexually active and of childbearing potential must use, with their partner, an approved method of highly effective contraception from the time of informed consent until 31 days after last IMP administration. This has been calculated based on 30 days (one female menstrual cycle) plus 5 half-lives of the study drug. Five half-lives has been calculated as 1 day.

The following highly effective methods are acceptable:

- Combined (oestrogen and progestogen-containing) hormonal contraception associated with inhibition of ovulation:
  - oral
  - intravaginal
  - transdermal
- Progestogen-only hormonal contraception associated with inhibition of ovulation
  - oral
  - injectable/implantable
  - intrauterine hormone-releasing system
- Intrauterine device
- Vasectomised partner
- Bilateral tubal occlusion

Female subjects are not required to use any of the above contraceptive methods if their sexual partner is female.

These contraception requirements are considered to be more conservative than the guidance issued by the Heads of Medicines Agency: Clinical Trials Facilitation Group [1].

All subjects (male and female subjects of childbearing potential)

Alternatively, sexual abstinence is considered a highly effective method only if defined as refraining from heterosexual intercourse during the entire period of risk associated with the study treatments. The reliability of sexual abstinence needs to be evaluated in relation to the duration of the clinical trial and the preferred and usual lifestyle of the subject.

Females of non-childbearing potential

Female subjects who are not of childbearing potential do not need to use any methods of contraception. A woman is considered of childbearing potential unless post-menopausal or permanently sterile. Permanent sterilisation methods include hysterectomy, bilateral salpingectomy and bilateral oophorectomy. A post-menopausal state is defined as no menses for 12 months without an alternative medical cause and confirmed by a follicular stimulating hormone result of ≥40 IU/L.

Sperm donation

Male subjects should not donate sperm for the duration of the study and for 91 days after last study drug administration.

Egg donation

Female subjects should not participate in egg donation from the time of informed consent, for the duration of the study until 31 days after last study drug administration.

References

1. Heads of Medicines Agency: Clinical Trials Facilitation Group. Recommendations related to contraception and pregnancy testing in clinical trials, version 1.1, September 21, 2020. Available at: https://www.hma.eu/fileadmin/dateien/Human_Medicines/01-About_HMA/Working_Groups/CTFG/2020_09_HMA_CTFG_Contraception_guidance_Version_1.1.pdf. Last accessed February 12, 2024.

Assessment of safety

Definition and classification of adverse events

An AE is any untoward medical occurrence in a subject that occurs either before dosing (referred to as a pre-dose AE) or once a medicinal product has been administered, including occurrences which are not necessarily caused by or related to that product.

An adverse drug reaction (ADR or treatment-related AE) is any AE where a causal relationship with the study drug is at least a reasonable possibility (possibly related or related).

AEs will be monitored from the time the subject signs the ICF until after the final follow-up call. The severity of AEs should be assessed as follows:

- Mild: An AE that is easily tolerated by the subject, causes minimal discomfort and does not interfere with everyday activities.
- Moderate: An AE that is sufficiently discomforting to interfere with normal everyday activities; intervention may be needed.
- Severe: An AE that prevents normal everyday activities; treatment or other intervention usually needed.

Assessment of causality

Every effort should be made by the investigator to try to explain each AE and assess its relationship, if any, to study drug. The temporal relationship of the event to study drug administration should be considered in the causality assessment (i.e. if the event starts soon after study drug administration and resolves when the study drug is stopped). Causality should be assessed using the following categories:

- Unrelated: Clinical event with an incompatible time relationship to study drug administration, and that could be explained by underlying disease or other drugs or chemicals or is incontrovertibly not related to the study drug.
- Possibly related: Clinical event with a reasonable time relationship to study drug administration, and that is unlikely to be attributed to concurrent disease or other drugs or chemicals.
- Related: Clinical event with plausible time relationship to study drug administration and that cannot be explained by concurrent disease or other drugs or chemicals.

The degree of certainty with which an AE is attributed to study drug administration (or alternative causes, e.g. natural history of the underlying disease, concomitant therapy) will be determined by how well the experience can be understood in terms of one or more of the following:

- Known pharmacology of the study drug
- Reactions of a similar nature have been previously observed with the IMP or this class of drug
- The experience being related by time to study drug administration, terminating with study drug withdrawal or recurring on re-challenge
- Alternative cause.

Recording adverse events

Adverse events (including serious AEs [SAEs]) will be recorded from the time of providing written informed consent until discharge from the study at the follow-up call. During each study visit, the subject will be questioned directly regarding the occurrence of any adverse medical event according to the schedule in the source. All AEs, whether ascribed to study procedures or not, will be documented immediately in the subject's source. This will include the date and time of onset, a description of the AE, severity, duration, actions taken, outcome and an investigator's current opinion on the relationship between the study drug and the event. A diagnosis and final opinion on the relationship between the study drug and the event will be provided at the end of the study by the investigator.

Any subject who withdraws from the study due to an AE will be followed up until the outcome is determined and written reports are provided by the investigator.

Serious adverse events

An SAE is defined as any untoward medical occurrence that at any dose:

- Results in death
- Is life-threatening
- Requires hospitalisation or prolongation of existing hospitalisation
- Results in persistent or significant disability or incapacity
- Consists of a congenital anomaly or birth defect
- An important medical event as recognised by the investigator.

Serious adverse events must be immediately reported to the sponsor.

Definition of suspected unexpected serious adverse reactions

Suspected unexpected serious adverse reactions (SUSARs) are AEs that are believed to be related to a study drug and are both unexpected (i.e. the nature or severity is not expected from the information provided in the Investigator Brochure or Summary of Product Characteristics) and serious. SUSARs are subject to expedited reporting to the UK Medicines and Healthcare products Regulatory Agency and ethics committee.

Analysis populations

Safety population

The safety population included all subjects who received at least one dose of study drug. The safety populations for Parts 1 and 2 of the study were defined separately. The safety analysis set was defined on a regimen basis and included all safety data from the subjects included in the safety population who had received that regimen.

Pharmacokinetic population

The pharmacokinetic population was defined separately for Parts 1 and 2, and included all subjects who received at least one dose of study drug and who satisfied the following criteria for at least one profile:

- No missing samples or invalid post-dose analytical results at critical time points, i.e. around C_{max}.
- No relevant protocol deviations that could have impacted the study objectives with respect to the pharmacokinetic endpoints.
- No relevant AEs such as vomiting that suggested that the whole dose was not available for absorption for a particular subject.

The PHARMACOKINETICS analysis set was defined on a per-treatment basis and included all relevant data from the subjects included in the pharmacokinetic population who received that regimen.

Pharmacodynamic population

The pharmacodynamic population, defined for Part 2 only, included all enrolled subjects who received at least one dose of study drug and who had a valid pre-dose analytical result for baseline, a minimum of one valid post-dose analytical result and who satisfied the following criteria:

- No relevant protocol deviations that could have impacted the study objectives with respect to the pharmacodynamic endpoints.
- No relevant adverse events, such as vomiting, which suggested that the whole dose was not absorbed for a particular subject.

The pharmacodynamic analysis set was defined on a regimen basis and included all pharmacodynamic data from the subjects included in the pharmacodynamic population who received that regimen.

Supplementary materials – results

Clinical laboratory evaluation – haematology, clinical chemistry and urinalysis

Part 1

Individual fluctuations were observed, with a small proportion of haematology and clinical chemistry values shifting from within the reference range at baseline (Period 1 Day 1, pre-dose) to outside the normal reference range after dosing with study drug for ≤2 subjects, with the following exceptions:

- 3 (20.0%) subjects who received ACM-001.1 followed by pindolol or the reverse sequence had: shifts in erythrocyte count to below the reference range at 24 hours after dosing with ACM-001.1 15 mg.
- 4 (26.7%) and 4 (28.6%) subjects had shifts in protein to below the reference range at 24 hours after dosing with ACM-001.1 15 mg and pindolol 30 mg, respectively.
- 4 (28.6%) subjects had shifts in calcium to below the reference range at 24 hours after dosing with pindolol 30 mg.

No subject experienced AEs associated with out-of-range haematology or clinical chemistry values following administration of ACM-001.1 or pindolol in Part 1.

No individual urinalysis results were considered clinically relevant or reported as TEAEs

Part 2

Individual fluctuations were observed, with a small proportion of haematology and clinical chemistry values shifting from within the reference range at baseline to outside the normal reference range after dosing with study drug. Shifts from within the reference range at baseline to outside the reference range after dosing were recorded for ≤2 subjects receiving any regimen, with the following exceptions:

- 3 (42.9%) subjects receiving ACM-001.1 5 mg had shifts in chloride to above the reference range at Day 2 pre-dose.
- 3 (42.9%) subjects receiving pindolol 20 mg had shifts in bicarbonate to below the reference range at Day 2 pre-dose.
- 4 (57.1%) subjects receiving pindolol 20 mg had shifts in protein to below the reference range at Day 2 pre-dose and Day 4 pre-dose.

- 3 (42.9%) subjects receiving ACM-001.1 15 mg had shifts in protein to below the reference range at day 2 pre-dose.
- 4 (57.1%) subjects receiving ACM-001.1 15 mg had shifts in calcium to below the reference range at Day 2 pre-dose.

One (14.3%) subject receiving ACM-001.1 15 mg experienced the mild TEAE of alanine aminotransferase (ALT) increased, which was considered to be possibly related to study drug. The subjects had an ALT value of 129 IU/L (normal reference range 10 to 50 IU/L for males), increased from baseline by 77 IU/L, at Day 5 24 hours post-final dose. The subject's ALT remained elevated at discharge (127 IU/L), but the TEAE was considered to have resolved at a follow-up visit (ALT value of 76 IU/L, 24 IU/L above baseline).

One (16.7%) subject receiving ACM-001.1 10 mg had elevated myoglobin (261 IU/L; normal reference range 28 IU/L to 72 IU/L) at Day 4 pre-dose. This elevation was associated with the mild TEAE of exacerbated foot cramps, which was considered to be possibly related to administration of study drug. The subject also had an elevation in creatine kinase at Day 4 pre-dose (916 IU/L; normal reference range 38 IU/L to 204 IU/L) alongside the elevated myoglobin. A diagnosis of rhabdomyolysis was made for this subject, which was considered mild and possibly related to administration of study drug. As this subject had already completed dosing, no further action was taken. The subject was considered to have recovered at a follow-up visit when serum creatine kinase was within the reference range and myoglobin was only marginally outside the reference range (value of 26 IU/L).

No individual urinalysis results were considered clinically relevant or reported as TEAEs.

Blood pressure and heart rate

Part 1

Several subjects experienced substantial decreases from baseline in diastolic BP (decrease of >10 mmHg). A higher number of decreases was noted for pindolol 30 mg, but there was no notable difference in the number of decreases between the ACM-001.1 15 mg and pindolol 15 mg groups. The highest number of substantial decreases in diastolic BP were noted at the following time points for each regimen:

- ACM-001.115 mg: 4 substantial decreases at 2 and 8 hours post-dose

- Pindolol 30 mg: 7 substantial decreases at 2 and 3 hours post-dose
- Pindolol 30 mg: 6 substantial decreases at 2.5, 8 and 12 hours post-dose
- Pindolol 30 mg: 5 substantial decreases at 4, 5, 6 and 10 hours post-dose
- Pindolol 15 mg: 4 substantial decreases at 6 hours post-dose.

The majority of these substantial changes were not associated with out-of-range values; however, several TEAEs of low blood pressure were reported for subjects who experienced decreases in systolic BP to <100 mmHg.

No substantial change (increase or decrease >20 mmHg) from baseline in systolic BP at any other time post-dose was observed.

Following dosing with ACM-001.1 15 mg, 4 subjects had a substantial increase in heart rate at 16 h post-dose; none of these was considered clinically significant or reported as a TEAE.

Part 2

Decreases from baseline (Day 1, pre dose of the corresponding study period; calculated from the arithmetic mean of the triplicate values) were reported at the majority of time points across all regimens for diastolic BP, and at several timepoints across all regimens for systolic BP:

- 4 subjects receiving ACM-001.1 15 mg had substantial decreases in diastolic BP at Day 1 2 h post-dose and Day 2, 2 h post-dose.

The majority of substantial changes were not associated with any out-of-range values; however, several TEAEs of decreased BP were reported for subjects who experienced systolic BP <100 mmHg.

There were no notable changes in mean HR parameters from baseline.

Electrocardiograms

Part 1

No clinically relevant differences between ACM-001.1 15 mg, pindolol l30 mg and pindolol 15 mg for any ECG parameter and no clinically relevant mean changes from baseline (Day 1, pre-dose of the corresponding study period) for any ECG parameter measured were observed.

One subject had a QTcF value of 453 msec following administration of ACM-001.1 15 mg and another had 3 QTcF values >450 msec (455, 456 and 464 msec at 1, 4 and 12 hours, respectively) following administration of pindolol 30 mg. No subject receiving pindolol 15 mg had a QTcF value >450 msec at any time point. No subject receiving ACM-001.1 15 mg or pindolol 15 mg had a QTcF increase ≥30 msec. With pindolol 30 mg, 1 subject at the 4 hours post-dose and 3 at 12 hours post-dose had a QTcF increase between 30 to 60 msec. However, the majority of these results were not associated with an out-of-range QTcF value. No individual ECG result was considered clinically significant and reported as a TEAE.

Part 2

No clinically relevant differences between dose levels of ACM-001.1 or between ACM-001.1 and pindolol for any ECG parameter and no notable mean changes from baseline (Day 1, pre-dose) for any ECG parameter measured were observed. No subject had a QTcF value > 450 msec at any post-dose time point, and none receiving ACM-001.1 5 mg and ≤1 receiving any other regimen had a QTcF increase of > 30 msec. The majority of these results were not associated with an out-of-range QTcF value, and no individual ECG result was considered clinically significant and reported as a TEAE.

Supplementary Table 1. Pharmacokinetic and pharmacodynamic sampling schedule

Part 1

Study day	Timepoint (hour)	Plasma pharmacokinetic samples	Urine pharmacokinetic sample collection
1	Pre-dose	X	>0–2
	0.33	X	
	0.66	X	
	1	X	
	1.5	X	
	2	X	
	2.5	X	>2–4
	3	X	
	4	X	
	5	X	>4–6
	6	X	
	8	X	>6–8
	10	X	>8–10
	12	X	>10–12
	16	X	>12–24
2	24	X	
3	48		X
4	Pre-dose	X	X
	0.33	X	>0–2
	0.66	X	
	1	X	
	1.5	X	
	2	X	
	2.5	X	>2–4
	3	X	

	4	X	
	5	X	>4–6
	6	X	
	8	X	>6–8
	10	X	>8–10
	12	X	>10–12
	16	X	>12–24
5	24	X	

Part 2

<table>
<tr><th>Study day</th><th>Timepoint (hour)</th><th>Plasma pharmacokinetic samples</th><th>Urine pharmacokinetic sample collection</th><th>Pharmacodynamics – bloods</th><th>Pharmacodynamics – heart rate and BP</th></tr>
<tr><td rowspan="15">1</td><td>Pre-dose</td><td>X</td><td rowspan="6">>0–2</td><td>X</td><td>X</td></tr>
<tr><td>0.33</td><td>X</td><td></td><td>X</td></tr>
<tr><td>0.66</td><td>X</td><td></td><td>X</td></tr>
<tr><td>1</td><td>X</td><td></td><td>X</td></tr>
<tr><td>1.5</td><td>X</td><td></td><td>X</td></tr>
<tr><td>2</td><td>X</td><td></td><td>X</td></tr>
<tr><td>2.5</td><td>X</td><td rowspan="3">>2–4</td><td></td><td>X</td></tr>
<tr><td>3</td><td>X</td><td></td><td>X</td></tr>
<tr><td>4</td><td>X</td><td></td><td>X</td></tr>
<tr><td>5</td><td>X</td><td rowspan="2">>4–6</td><td></td><td>X</td></tr>
<tr><td>6</td><td>X</td><td></td><td>X</td></tr>
<tr><td>8</td><td>X</td><td>>6–8</td><td></td><td>X</td></tr>
<tr><td>10</td><td>X</td><td>>8–10</td><td></td><td>X</td></tr>
<tr><td>12</td><td>X</td><td>>10–12</td><td></td><td>X</td></tr>
<tr><td>14</td><td>X</td><td rowspan="2">>12–24</td><td></td><td></td></tr>
<tr><td>2</td><td>24/pre-dose</td><td>X</td><td></td><td>X</td></tr>
<tr><td>3</td><td>48/pre-dose</td><td></td><td></td><td></td><td></td></tr>
<tr><td rowspan="5">4</td><td>Pre-dose</td><td>X</td><td></td><td>X</td><td>X</td></tr>
<tr><td>0.33</td><td>X</td><td rowspan="4">>0–2</td><td></td><td>X</td></tr>
<tr><td>0.66</td><td>X</td><td></td><td>X</td></tr>
<tr><td>1</td><td>X</td><td></td><td>X</td></tr>
<tr><td>1.5</td><td>X</td><td>X</td><td>X</td></tr>
</table>

	2	X			X
	2.5	X	>2–4		X
	3	X			X
	4	X			X
	5	X	>4–6		X
	6	X			X
	8	X	>6–8		X
	10	X	>8–10		X
	12	X	>10–12		X
	14		>12–24		
5	24	X			X
	36		X		
6	48	X	X		X
7	72		X		

Supplementary Table 2. Relative bioavailability of S-pindolol following single and multiple doses of ACM-001.1 5, 10 and 15 mg and pindolol 20 mg (pharmacokinetic analysis set)

		ACM-001.1		Pindolol 20 mg			
Comparison	Pharmacokinetic parameter	n	Adjusted geometric mean	n	Adjusted geometric mean	Ratio (%)*	90% CI†
Day 1							
ACM-001.1 5 mg vs pindolol 20 mg	C_{max} (ng/mL)	7	23.8	7	40.4	58.96	45.38, 76.61
	$AUC_{(0–tau)}$ (ng.h/mL)	7	129	7	216	59.42	43.98, 80.27
ACM-001.1 10 mg vs pindolol 20 mg	C_{max} (ng/mL)	6	41.1	7	40.4	101.76	77.49, 133.64
	$AUC_{(0–tau)}$ (ng.h/mL)	6	227	7	216	105.05	76.80, 143.68
ACM-001.1 15 mg vs pindolol 20 mg	C_{max} (ng/mL)	7	73.6	7	40.4	182.49	140.45, 237.11
	$AUC_{(0–tau)}$ (ng.h/mL)	7	414	7	216	191.44	141.70, 258.66
Day 4							
ACM-001.1 5 mg vs pindolol 20 mg	C_{max} (ng/mL)	7	27.0	7	48.2	56.15	43.21, 72.95
	$AUC_{(0–tau)}$ (ng.h/mL)	7	159	7	293	54.32	40.20, 73.39
ACM-001.1 10 mg vs pindolol 20 mg	C_{max} (ng/mL)	6	50.3	7	48.2	104.35	79.46, 137.05
	$AUC_{(0–tau)}$ (ng.h/mL)	6	294	7	293	100.11	73.19, 136.93
	C_{max} (ng/mL)	7	80.5	7	48.2	167.22	128.70, 217.27

ACM-001.1 15 mg vs pindolol 20 mg	$AUC_{(0–tau)}$ (ng.h/mL)	7	480	7	293	163.48	121.00, 220.87

Results obtained from mixed effects model of natural log transformed pharmacokinetic parameters including terms for treatment, day and a treatment by day interaction term fitted as fixed effects and subject as a random effect

$AUC_{(0-t)}$, area under the plasma concentration-time curve from zero to the time of the last quantifiable concentration; C_{max}, maximum plasma concentration

*Ratio of adjusted geometric means with comparison presented as test/reference

†CI, confidence interval for the ratio of adjusted geometric means

Supplementary Table 3. Dose proportionality of S-pindolol following single doses of pindolol 30 mg versus 15 mg (pharmacokinetic analysis set)

		Pindolol 30 mg		Pindolol 15 mg			
Comparison	Pharmacokinetic parameter	N	Adjusted geometric mean	N	Adjusted geometric mean	Ratio (%)*	90% CI†
Pindolol 30 mg vs pindolol 15 mg	C_{max}/D (ng/mL/mg)	15	2.30	8	2.62	88.01	(72.61, 106.68)
	$AUC_{(0-t)}$/D (ng.h/mL/mg)	14	13.8	8	15.0	91.86	(70.92, 118.99)
	$AUC_{(0-inf)}$/D (ng.h/mL/mg)	14	14.1	8	15.3	91.99	(70.50, 120.03)

Results obtained from an ANOVA model of natural log transformed dose corrected pharmacokinetic parameters including terms for regimen fitted as fixed effect

$AUC_{(0-t)}$, area under the plasma concentration-time curve from zero to the time of the last quantifiable concentration; C_{max}, maximum plasma concentration; D, dose

*Ratio of adjusted geometric means with comparison presented as test/reference

†CI, confidence interval for the ratio of adjusted geometric means

Supplementary Table 4. **Geometric mean** cumulative excretion and recovery of S-pindolol, R-pindolol and pindolol in urine following a single dose of ACM-001.1 or pindolol (pharmacokinetic analysis set)

	S-pindolol		R-pindolol		Pindolol	
Treatment	CumAe, **ng (CV%)**	Cum%Ae, **% (CV%)**	CumAe, **ng (CV%)**	Cum%Ae, **% (CV%)**	CumAe, **ng (CV%)**	Cum%Ae, **% (CV%)**
ACM-001.1 15 mg (n=15)	4,030,000 (30.9%)	26.891 (30.9%)	1,300 (111.4%)	0.0009 (111.4%)	4,030,000 (30.9%)	26.899 (30.9%)
Pindolol 15 mg (n=8)	2,400,000 (15.7%)	16.027 (15.7%)	2,090,000 (20.8%)	13.911 (20.8%)	4,490,000 (17.6%)	29.938 (17.6%)
Pindolol 30 mg (n=15)	3,580,000 (18.5%)	11.943 (18.5%)	3,000,000 (29.4%)	9.996 (29.4%)	6,580,000 (23.0%)	21.939 (23.0%)

CumAe, cumulative amount of drug excreted unchanged in urine; Cum%Ae, cumulative percentage of drug excreted unchanged in urine; **CV%, coefficient of variation (%)**

Supplementary Table 5. **Geometric mean** cumulative excretion and recovery of S-pindolol, R-pindolol and pindolol in urine following multiple doses of ACM-001.1 or pindolol (pharmacokinetic analysis set)

	S-pindolol		R-pindolol		Pindolol	
Treatment	CumAe, **ng (CV%)**	Cum%Ae, **% (CV%)**	CumAe, **ng (CV%)**	Cum%Ae, **% (CV%)**	CumAe, **ng (CV%)**	Cum%Ae, **% (CV%)**
			Day 1			
ACM-001.1 5 mg (n=7)	359,000 (150.3%)	7.186 (150.3%)	NC	NC	NC	NC
ACM-001.1 10 mg (n=6)	2,140,000 (58.6%)	21.145 (58.6%)	NC	NC	NC	NC
ACM-001.1 15 mg (n=7)	3,570,000 (28.7%)	10.843 (28.5%)	NC	NC	NC	NC
Pindolol 20 mg (n=7)	2,170,000 (28.5%)	10.833 (28.5%)	1,920,000 (46.5%)	9.617 (46.5%)	4,090,000 (36.6%)	20.452 (36.6%)
			Day 4			
ACM-001.1 5 mg (n=7)	877,000 (68.8%)	17.539 (68.8%)	NC	NC	NC	NC
ACM-001.1 10 mg (n=6)	2,700,000 (42.2%)	27.025 (42.4%)	NC	NC	NC	NC
ACM-001.1 15 mg (n=7)	4,000,000 (43.4%)	26.685 (43.4%)	NC	NC	NC	NC
Pindolol 20 mg (n=7)	2,870,000 (35.2%)	14.345 (35.2%)	2,530,000 (46.9%)	12.643 (46.9%)	5,400,000 (40.4%)	26.987 (40.4%)

CumAe, cumulative amount of drug excreted unchanged in urine; Cum%Ae, cumulative percentage of drug excreted unchanged in urine; **CV%, coefficient of variation (%);** NC, not calculated

Supplementary Table 6. Mean serum biomarker concentrations **(mean change from baseline)** in serum biomarker concentrations from baseline following administration of multiple oral doses of ACM-001.1 or pindolol (pharmacodynamic analysis set)

	Pindolol 20 mg (n=7)	ACM-001.1 5 mg (n=7)	ACM-001.1 10 mg (n=6)	ACM-001.1 15 mg (n=7)
DHEA/cortisol (ng/mL)				
Day 1 pre-dose	154.70 (NA)	134.66 (NA)	143.27 (NA)	118.64 (NA)
Day 4 pre-dose	145.46 (−9.24)	129.64 (−5.01)	106.35 (−36.92)	114.57 (−3.35) (n=6)
Day 4 1.5 hours	90.33 (−64.37)	97.41 (−37.24)	74.55 (−68.72)	85.42 (−33.21)
Myostatin (pg/mL)				
Day 1 pre-dose	69,990.9 (NA)	17,697.96 (NA)	145,071.7 (NA)	10,545.8 (NA)
Day 4 pre-dose	79,972.3 (−1,423.7) (n=6)	19,351.4 (1,653.9)	142,416.7 (−2,655.0)	14,908.8 (2,810.3) (n=6)
Day 4 1.5 hours	62,566.6 (−7,424.3)	20,063.6 (2,366.0)	152,103.3 (7,031.7)	10,595.6 (49.7)
IGF1 (pg/mL)				
Day 1 pre-dose	1,987.9 (NA)	1,694.3 (NA)	1,456.2 (NA)	1,583.3 (NA)
Day 4 pre-dose	2,162.9 (175.0) (n=6)	1,852.9 (158.6)	1,102.8 (−353.3)	1,349.5 (−287.7) (n=6)
Day 4 1.5 hours	2,100.4 (112.6)	1,661.7 (−32.6)	1.096.2 (−360.0)	1444.7 (−138.6)
Folistatin (pg/mL)				
Day 1 pre-dose	12,716.7 (NA) (n=6)	13,941.7 (NA) (n=6)	14,250.0 (NA) (n=5)	11,266.7 (NA) (n=6)
Day 4 pre-dose	13,256.7 (640.0) (n=6)	14,155.0 (213.3) (n=6)	15,156.0 (806.0) (n=5)	9.420.0 (620.0) (n=5)

	Pindolol 20 mg (n=7)	ACM-001.1 5 mg (n=7)	ACM-001.1 10 mg (n=6)	ACM-001.1 15 mg (n=7)
Day 4 1.5 hours	13,142.0 (422.0) (n=5)	15,015.0 (1,073.3) (n=6)	15,012.0 (662.0) (n=5)	11,625.0 (358.3) (n=6)
PIIINP (pg/mL)				
Day 1 pre-dose	3,806.9 (NA)	2,625.7 (NA)	3,600.0 (NA)	2,675.7 (NA)
Day 4 pre-dose	3,644.9 (−162.0)	2,621.4 (−4.3)	4,125.0 (525.0)	2,595.0 (−73,3) (n=6)
Day 4 1.5 hours	3,523.6 (−283.3)	2,674.3 (48.6)	3,988.3 (388.3)	2,514.3 (−161.4)
MIG/CXCL9 (leptin) (pg/mL)				
Day 1 pre-dose	33.14 (NA)	32.93 (NA)	40.98 (NA)	34.63 (NA)
Day 4 pre-dose	32.61 (−0.53)	34.17 (1.24)	41.87 (0.88)	34.45 (−0.50) (n=6)
Day 4 1.5 hours	32.34 (−0.90)	33.93 (1.00)	40.10 (−0.88)	33.21 (−1.41)
ENA78 (pg/mL)				
Day 1 pre-dose	1,424.6 (NA)	1,445.1 (NA)	868.7 (NA)	1,934.9 (NA)
Day 4 pre-dose	1,408.6 (−16.0)	934.3 (−510.9)	828.3 (−40.3)	2,296.7 (138.0) (n=6)
Day 4 1.5 hours	1,294.0 (−130.6)	900.7 (−544.4)	782.0 (−86.7)	1,679.7 (−255.1)
Ghrelin (pg/mL)				
Day 1 pre-dose	385.1 (NA)	482.7 (NA)	245.2 (NA)	518.1 (NA)
Day 4 pre-dose	351.4 (−33.7)	371.7 (−111.0)	237.5 (−7.7)	348.0 (−139.8) (n=6)
Day 4 1.5 hours	295.0 (−130.6)	282.7 (−200.0)	191.3 (−53.8)	270.6 (−247.6)

	Pindolol 20 mg (n=7)	ACM-001.1 5 mg (n=7)	ACM-001.1 10 mg (n=6)	ACM-001.1 15 mg (n=7)
GHRH (pg/mL)				
Day 1 pre-dose	4.169 (NA)	6.493 (NA)	4.218 (NA)	7.546 (NA)
Day 4 pre-dose	4.361 (0.193)	9.417 (2.979)	4.618 (0.400)	5.413 (−0.323) (n=6)
Day 4 1.5 hours	4.563 (0.394)	9.076 (2.637)	5.460 (1.242)	7.310 (−0.236)
Somatostatin (pg/mL)				
Day 1 pre-dose	114.72 (NA)	114.69 (NA)	47.44 (NA)	67.65 (NA)
Day 4 pre-dose	98.50 (−16.21)	98.47 (−16.21)	57.19 (9.75)	68.70 (−8.92) (n=6)
Day 4 1.5 hours	116.74 (−15.80) (n=6)	76.05 (−38.64)	43.03 (−4.41)	60.19 (−7.46)

CXCL9, chemokine (C-X-C motif) ligand 9; DHEA, dehydroepiandrosterone; ENA78, C-X-C motif chemokine 5; GHRH, growth hormone releasing hormone; IGF1, insulin-like growth factor 1; MIG, monokine induced by gamma interferon; NA, not applicable; PIIINP, procollagen 3 N-terminal peptide.

Supplementary Table 7. Spirometry (safety analysis set)

Regimen	Day	n	Mean (SD)	Median (min, max)	Absolute change from baseline Mean (SD)	Absolute change from baseline Median (min, max)	Change from baseline (%) Mean (SD)	Change from baseline (%) Median (min, max)
FEV$_1$ (L)								
Pindolol 20 mg	1	7	3.544 (0.346)	3.550 (3.04, 4.13)				
	4	7	3.554 (0.443)	3.470 (3.04, 4.28)	0.010 (0.160)	0.000 (−0.25, 0.23)	0.127 (4.439)	0.000 (−7.35, 6.12)
ACM-001.1 5 mg	1	7	3.530 (0.638)	3.340 (2.79, 4.37)				
	4	7	3.504 (0.737)	3.150 (2.70, 4.51)	−0.02(0.141)	−0.040 (−0.23, 0.17)	− 1.112 (3.780)	−1.370 (−6.89, 3.98)
ACM-001.1 10 mg	1	6	3.965 (0.695)	3.870 (3.07, 4.89)				
	4	6	4.005 (0.644)	4.080 (3.03, 4.83)	0.040 (0.216)	0.000 (−0.25, 0.38)	1.277 (5.290)	−0.084 (−5.39, 9.60)
ACM-001.1 15 mg	1	7	3.923 (1.165)	3.920 (2.48, 5.61)				
	4	7	3.904 (1.101)	3.920 (2.52, 5.47)	−0.01 (0.090)	0.000 (−0.15, 0.06)	−0.105 (1.960)	0.000 (−3.02, 1.83)
FVC (L)								
Pindolol 20 mg	1	7	4.413 (0.572)	4.250 (3.93, 5.49)				
	4	7	4.396 (0.774)	4.140 (3.63, 5.85)	−0.01 (0.223)	−0.050 (−0.36, 0.36)	−0.833 (4.865)	−1.171 (−9.02, 6.56)
ACM-001.1 5 mg	1	7	4.309 (0.712)	4.540 (3.27, 5.18)				
	4	7	4.197 (0.779)	4.180 (3.20, 5.30)	−0.11 (0.213)	−0.070 (−0.37, 0.12)	−2.712 (4.759)	−2.141 (−8.33, 2.40)
ACM-001.1 10 mg	1	6	4.878 (0.868)	5.035 (3.55, 5.74)				

Regimen	Day	n	Mean (SD)	Median (min, max)	Absolute change from baseline Mean (SD)	Absolute change from baseline Median (min, max)	Change from baseline (%) Mean (SD)	Change from baseline (%) Median (min, max)
	4	6	4.953 (0.945)	5.025 (3.48, 5.95)	0.075 (0.184)	0.160 (−0.23, 0.22)	1.377 (3.741)	3.139 (−4.55, 4.31)
ACM-001.1 15 mg	1	7	4.630 (1.566)	4.650 (2.67, 6.76)				
	4	7	4.633 (1.474)	4.750 (2.96, 6.63)	0.003 (0.157)	−0.010 (−0.16, 0.29)	0.795 (4.819)	−0.274 (−3.23, 10.8)
FEV_1/FVC (%)								
Pindolol 20 mg	1	7	80.7 (4.3)	82.0 (75, 85)				
	4	7	81.6 (4.5)	83.0 (73, 87)	0.9 (2.0)	1.0 (−2, 3)	1.075 (2.510)	1.220 (−2.67, 3.90)
ACM-001.1 5 mg	1	7	82.0 (5.5)	84.0 (70, 86)				
	4	7	83.1 (4.9)	84.0 (74, 89)	1.1 (2.3)	1.0 (−2, 4)	1.482 (2.916)	1.190 (−2.44, 5.71)
ACM-001.1 10 mg	1	6	81.8 (9.6)	82.0 (69, 97)				
	4	6	81.8 (10.6)	79.0 (73, 100)	0.0 (3.6)	0.5 (−6, 4)	−0.004 (4.615)	0.581 (−7.41, 5.80)
ACM-001.1 15 mg	1	7	85.9 (4.6)	84.0 (80, 93)				
	4	7	85.3 (4.2)	84.0 (80, 91)	−0.6 (3.5)	0.0 (−8, 3)	− 0.581 (3.816)	0.000 (−8.60, 3.41)

FVC, forced vital capacity; FEV, forced expiratory volume

Supplementary figure 1. Heart rate on Day 4 with racemic pindolol 20 mg and ACM-001-1 5, 10 and 15 mg at steady state (Part 2, pharmacodynamic analysis set)

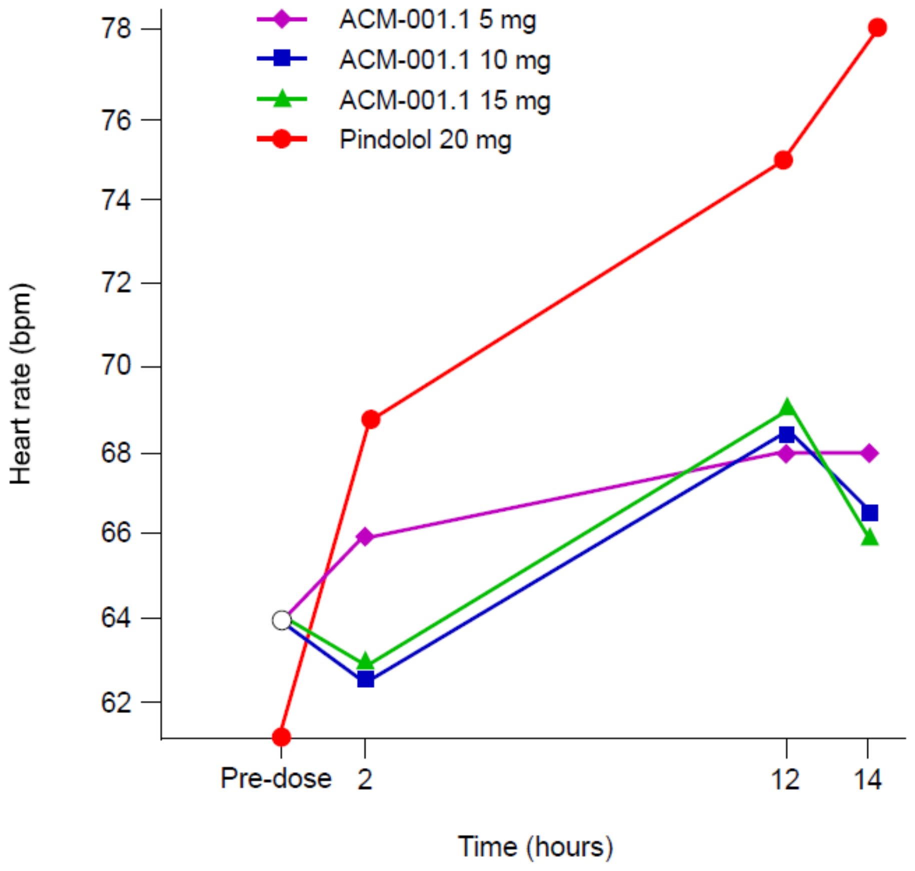

Supplement: Supplementary file 2 — Figure S1. Heart rate on Day 4 with racemic pindolol 20 mg and ACM‐001‐1 5, 10 and 15 mg at steady state (Part 2, pharmacodynamic analysis set) [file JCSM-16-e13651-s001.pdf]
